# Fracture guide wire during an elective percutaneous coronary intervention

**DOI:** 10.1002/ccr3.964

**Published:** 2017-04-20

**Authors:** George Samanidis, Constantinos Contrafouris, Theofani Antoniou, Konstantinos Perreas

**Affiliations:** ^1^ First Department of Adult Cardiac SurgeryOnassis Cardiac Surgery CenterAthensGreece; ^2^ Department of Paediatric and CongenitalOnassis Cardiac Surgery CenterAthensGreece; ^3^ Department of AnesthesiologyOnassis Cardiac Surgery CenterAthensGreece

**Keywords:** Coronary angiography, guide wire fracture, PCI

## Abstract

Dose the fractured guide wire should be removed during or after percutaneous coronary interventions? In case when the patients have unstable hemodynamic status the fractured guide wire should be removed with percutaneous or surgical method. Antiplatelet drug administration should be considered after procedure to prevention of the coronary artery thrombosis.

A 65‐year‐old male was referred to our department and was scheduled to coronary artery bypass grafting (CABG). During preoperative assessment on the coronary angiography a foreign body revealed in the OM_1_ (Fig. 1A–C) and on the chest X‐ray (Fig. 1D). During an elective CABG, the fractured guide wire (FGW) was removed without difficulty from OM_1_ (Fig. 2A, B, D). As for the LAD, closed endarterectomy (atheroma with stent) (Fig. 2C and D) was performed. Although the FGW is a rare complication during PCI the residual segment should be removed for prevention of the life treating complications 1, 2. We present in the Figure 3 algorithm for managing guide wire fractured during PCI.

**Figure 1:**
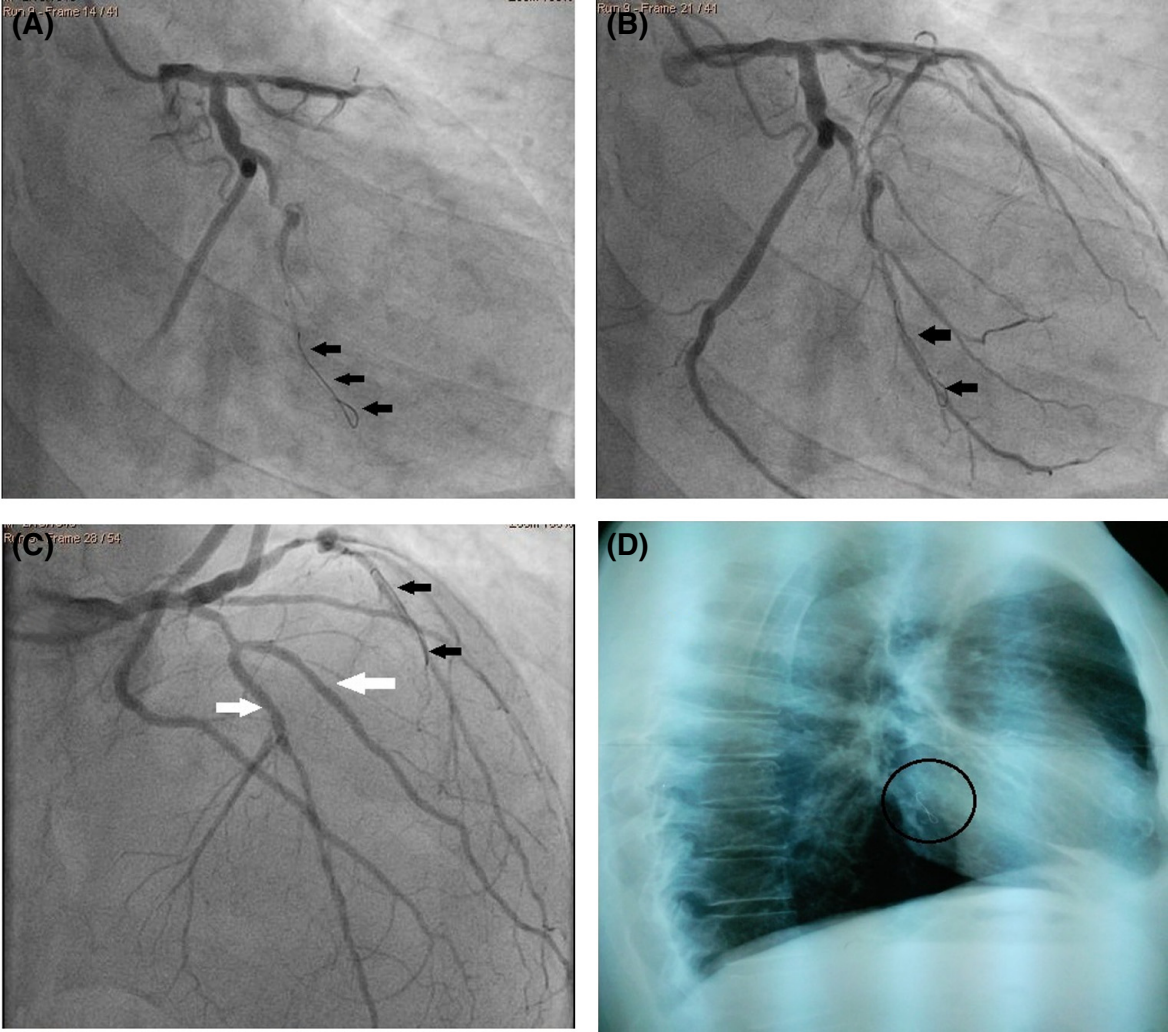
Coronary angiography and Chest radiography: (A and B) Coronary angiography: fractured guide wire in the first obtuse marginal branch (OM_1_) (black arrow), (C) Coronary angiography: fractured guide wire in the first obtuse marginal branch (OM_1_) (black arrow) and with white arrow noted the left anterior descending artery (LAD) and the first diagonal branch (D_1_). (D) Chest radiography (profile view) demonstrates a fractured guide wire in the black circle.

**Figure 2:**
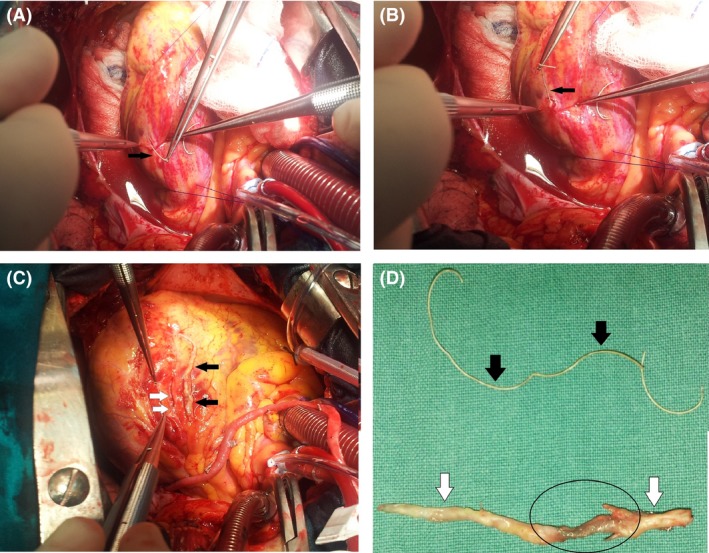
Intraoperative views: (A and B) Fractured guide wire in the first obtuse marginal branch (OM_1_) (black arrow), (C) Endarterectomy in the left anterior descending artery (white arrow) and atheroma (black arrow). (D) Fractured guide wire (black arrow). Atheroma which removed from the left anterior descending artery (white arrow) and the stent in the atheroma (black circle).

**Figure 3:**
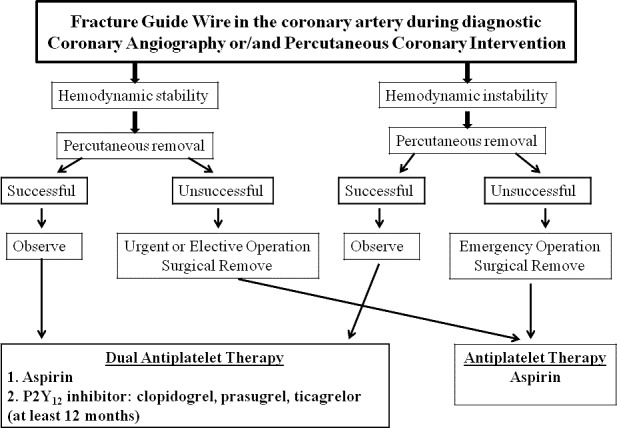
Algorithm for managing a guide wire fractured in the coronary artery.

## Authorship

GS, CC, TA and KP: took part in the care of the patient and contributed to the medical literature search. All authors approved the final manuscript.

## Conflict of Interest

None declared.
